# Positive contribution of hydroxytyrosol-enriched wheat bread to HbA_1_c levels, lipid profile, markers of inflammation and body weight in subjects with overweight/obesity and type 2 diabetes mellitus

**DOI:** 10.1007/s00394-023-03133-9

**Published:** 2023-04-05

**Authors:** Panagiota Binou, Athena Stergiou, Ourania Kosta, Nikolaos Tentolouris, Vaios T. Karathanos

**Affiliations:** 1 Laboratory of Chemistry-Biochemistry-Physical Chemistry of Foods, Department of Nutrition and Dietetics, Harokopio University of Athens, 70 El. Venizelou Ave, 17671 Athens, Greece; 2 1st Department of Propaedeutic and Internal Medicine, Diabetes Center, Medical School, National and Kapodistrian University of Athens, Laiko General Hospital, 75 Mikras Asias Str, 11527 Athens, Greece

**Keywords:** Glucose, Insulin, HbA_1_c, Inflammatory markers, Cholesterol, Body weight

## Abstract

**Purpose:**

The aim of the present study was to assess the impact of the daily consumption of bread enriched with hydroxytyrosol on HbA_1_c and blood lipid levels, inflammatory markers and weight loss.

**Methods:**

Sixty adults with overweight/obesity and type 2 diabetes mellitus (29 male, 31 female) participated in a 12-week dietary intervention based on the Mediterranean diet and consumed daily 60 g of conventional whole wheat bread (WWB) or whole wheat bread enriched with hydroxytyrosol (HTB). Anthropometric characteristics were measured and venous blood samples were collected at baseline and at the end of the intervention.

**Results:**

Both groups experienced significant weight loss, body fat and waist circumference decrease (*p* < 0.001). Nonetheless, a greater body fat mass decrease was observed in the HTB group compared to the WWB group (14.4 ± 1.6 vs 10.2 ± 1.1%, *p* = 0.038). Significant reductions were also reported in fasting glucose, HbA_1_c and blood pressure in both groups (*p* < 0.05). Regarding glucose and HbA_1_c, greater decreases were observed in the intervention group (101.4 ± 19.9 vs. 123.2 ± 43.4 mg/dL, *p* = 0.015 and 6.0 ± 0.6 vs. 6.4 ± 0.9%, *p* = 0.093, respectively). At HTB group, significant reductions in blood lipid, insulin, TNF-αand adiponectin levels (*p* < 0.05) and a marginally significant reduction in leptin levels (*p* = 0.081) were also reported.

**Conclusion:**

Enrichment of bread with HT resulted in significant body fat mass reduction and positive effects on fasting glucose, insulin and HbA_1_c levels. It also contributed to reductions in inflammatory markers and blood lipid levels. Incorporation of HT in staple foods, like bread, may improve their nutritional profile and, in terms of a balanced diet, may contribute to the management of chronic diseases.

**Trial registration:**

The study was prospectively registered in clinicaltrials.gov (24th May 2021). ClinicalTrials.gov Identifier: NCT04899791.

## Introduction

Diabetes is a major health problem, which prevalence has reached alarming levels globally, with more than half a billion people been diagnosed worldwide. The prevalence is estimated to increase to 783 million by 2045, with induced health and socioeconomic costs [1]. Type 2 diabetes mellitus (T2DM), the most common type of diabetes, is characterized by chronic hyperglycaemia, which is the main pathological mechanism leading to organ and tissue damages and long term complications. The severity of chronic hyperglycaemia induced complications is affected by concomitant diseases (e.g. hypertension and dyslipidaemia) [2, 3]. Patients with T2DM typically show abnormalities in lipid metabolism, such as higher levels of small dense LDL particles and triglycerides and lower levels of HDL cholesterol. Cardiovascular disease (CVD), the most important complication of T2DM, is the most prevalent cause of morbidity and mortality in diabetic populations [4].

According to the World Health Organization (WHO), a healthy diet and maintenance of a normal body weight is a fundamental practice for prevention or delay of the onset of T2DM [5]. Among various dietary patterns, Mediterranean diet (MD) has been proved to reduce CVD in the diabetic sub-cohort of the PREDIMED study [6]. Anti-inflammatory effects and reduced oxidative stress are the most likely explanations for the observed protection of this dietary pattern rich in high-unsaturated fat [7].

Olive oil, the key culinary lipid ingredient of MD, contains abundant bioactive compounds such as monounsaturated fatty acids (MUFAs) and polyphenols. Among them, hydroxytyrosol (HT) stands out as a potent polyphenol with significant anti-oxidant effects; it reduces inflammation, production of oxidized low-density lipoprotein (oxLDL) and platelet aggregation, while it seems to exert insulin-like and insulin sensitizing effects [8–10]. For its health-promoting outcomes, European Food Safety Authority (EFSA) has authorized a health claim for HT, according to which, use of HT (5 mg/day) or its derivatives can offer protection to LDL particles from oxidative damage [11].

The major obstacle of the therapeutic use οf HT when consumed from natural sources is its low bioactivity. HT is well absorbed via passive transport in the small bowel and the colon; however, it has a rapid and intense metabolism resulting in very low concentrations in plasma [12, 13]. Besides that, HT is particularly susceptible to light, oxygen, heat and pH, environmental factors that lead to its degradation [14]. Microencapsulation of unstable bioactive compounds arises as a non-pharmaceutical strategy for the management of T2DM promoting the development of novel functional foods with negligible side effects [15]. HT is generally recognized as safe (GRAS) as a food ingredient, by EFSA and the Food and Drug Administration (FDA) [16, 17].

Given that wheat bread is a staple food and the most common carbohydrate-rich food consumed in the world [18], it becomes an excellent choice to be fortified with HT, so its nutritional value could be increased. To overcome the barriers regarding HT instability, its inclusion in a protective wall material constitutes an effective approach. Among various carriers, α-cyclodextrin is a soluble dietary fiber with positive postprandial glucose effects and is classified as “ADI not specified” (ADI = allowed daily intake) [19, 20].

The present study describes a three-month randomized dietary intervention addressed to patients with T2DM. The aim of the present study was to evaluate the long-term metabolic effects of the daily consumption of whole wheat HT-enriched bread. Biochemical parameters related to the disease, markers of inflammation as well as body weight, body composition and energy balance were examined.

## Materials and methods

### Subjects and study design

The target population of the study included individuals with overweight/obesity (body mass index (BMI) ≥ 25 kg m^−2^) and T2DM (glycosylated hemoglobin (HbA_1_c) < 8.5%), aged between 40 and 75 years, with normal exercise, eating and drinking habits. More specifically, subjects should not have been diagnosed with eating disorders (e.g. bulimia or anorexia nervosa, binge eating disorder) or be professional athletes. Additionally, individuals should have stable oral medication (hypoglycemic, hypolipidemic and antihypertensive) for the last three months before screening. The recruitment of the subjects took place in the diabetes clinic of the 1st Department of Propaedeutic and Internal Medicine, Laiko General Hospital, Athens University Medical School.

Exclusion criteria were history of cardiovascular, gastrointestinal, renal and endocrinological diseases, treatment for weight reduction and history of drug and/or alcohol abuse or psychiatric disease prohibiting adherence to the protocol. Eligible subjects were enrolled after being fully informed about the nature and procedures of the study and giving their written consent for participation.

The protocols were reviewed and approved by both the Institutional Review Board/Ethics Committee of Laiko General Hospital and the Harokopion University of Athens. The study protocol registration number is ClinicalTrials.gov: NCT04899791.

**Fig. 1:**
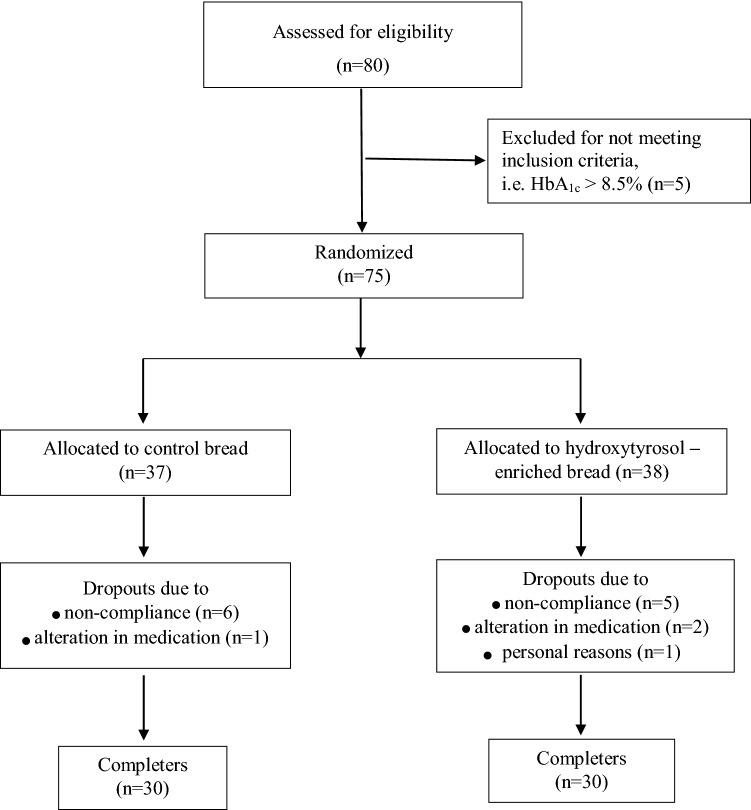
Flow chart of the study

A 12-week, single-blinded, randomized dietary intervention was conducted at the 1st Department of Propaedeutic and Internal Medicine, Laiko General Hospital, Medical School, National and Kapodistrian University of Athens, in collaboration with the Laboratory of Chemistry, Biochemistry and Physical Chemistry of Foods, Department of Nutrition and Dietetics, Harokopion University, Athens, Greece. A total of sixty subjects (n = 31 females, n = 29 males) participated (Fig. 1).

Before randomization, there was a run-in period that lasted two weeks. During these weeks, the subjects were trained in important parameters of the protocol (i.e. meal planning, portion sizes, counselling on the principles of the MD, completion of the 3-day weighed dietary records). After run-in period, the participants were randomized into two study groups to consume either whole wheat bread enriched with hydroxytyrosol (HTB), which contained 54 mg of HT per 100 g, or matching placebo [conventional whole wheat bread (WWB)]. Volunteers in both groups received weekly dietary counseling sessions by trained dietitians and were asked not to change their exercise habits during the study. A caloric deficit of 500 kcal of their daily energy requirements using an individualized dietary strategy was prescribed. The macronutrient distribution of the prescribed diets was as follows: 45% of energy as carbohydrates, 15–20% as proteins and 35–40% as fat. The participants were instructed to follow the dietary regime for 12 consecutive weeks and were advised to consume two slices of bread per day (1 slice = 30 g). The daily consumption of HT in the intervention group was 32.5 mg. The bread was supplied to the participants at each visit.

Total daily energy intake was calculated based on baseline measurements of weight and resting metabolic rate (RMR), multiplied with a physical activity level (PAL) of 1.4, which indicates a low level of physical activity. An energy intake of 1400–1800 kcal/day was calculated for the female and of 1800–2500 for the male participants. In this context, volunteers received weekly individualized diet plans with amounts of foods expressed in grams, in both raw and cooked terms, to facilitate comprehension and adherence. Adherence to the dietary intervention was evaluated in each visit by measurement of weight loss and completion of a 3-day weighed dietary record (two weekdays and one weekend day). Participants who did not follow at least 75% of the prescribed diets for two successive weeks were excluded from the study.

Demographic information, medical history, body weight history and energy intake [via a 24-h dietary recall and a semi-quantitive food frequency questionnaire (FFQ)] and expenditure data [via the International Physical Activity Questionnaire (IPAQ)] were collected at the baseline screening visit of the volunteers. The 24-h dietary recall, FFQ and IPAQ were also completed at the end of the 12-week study period.

During the first visit volunteers presented to the laboratory in the morning at 8.00 am after a 12-h overnight abstinence from food, fluid and exercise. Premenopausal female subjects were also asked not to arrange their visits on the menses phase of their cycle. A detailed clinical examination and anthropometric measurements, including body weight (electronic scale—TANITA WB-110MA, Japan), body composition (bioelectrical impedance analysis—Tanita BC-418, Tokyo, Japan), height (stadiometer—Seca Mode 220, Hamburg, Germany), waist circumference, blood pressure and RMR measurements, were performed at the first and last session. RMR was assessed by indirect calorimetry (Fitmate TM Cosmed, Rome, Italy). For the indirect calorimetry assessment which lasted 15 min, the first five minutes of each test were discarded and the assessment continued until there was a period of 5 consecutive minutes with a coefficient of variation of RMR ≤ 10%.

### Blood analyses

Fasting blood samples were collected at the first and the last session in pre-cooled vacutainers with K_3_EDTA as anticoagulant and centrifuged immediately (3000 rpm for 10 min at 4 °C) for plasma separation. For serum, blood was collected in plain vacutainers, allowed to clot at room temperature for 30 min and then centrifuged (3000 rpm for 10 min at 4 °C). After isolation, plasma and serum were stored at − 80 °C until analysis.

Measurement of HbA_1_c and basal biochemical measurements including plasma glucose, total cholesterol (TC), high density lipoprotein cholesterol (HDL-C), triacylglycerols (TAG), alanine aminotransferase (ALT), aspartateaminotransferase (AST), γ-glutamyl transferase (γ-GT), urea, creatinine, uric acid and total proteins were performed in serum, on an automated biochemical analyzer (Medilyzer), using commercially available diagnostic kits. Low density lipoprotein cholesterol (LDL-C) was calculated using Friedewald’s formula.

In plasma, leptin was assayed by a sandwich ELISA method on a microtiter plate using a commercially available human leptin kit (Human Leptin ELISA kit; Thermofisher, Waltham, MA, USA). C-reactive protein (CRP) was also determined by a sandwich ELISA method using a commercially available high-sensitivity kit (Human hsC-Reactive Protein/CRP ELISA kit; R&D systems, Minneapolis, MN, USA) and adiponectin, Tumor Necrosis Factor-α (TNF-α) and oxidized LDL by the following kits (Human Adiponectin ELISA kit; Elabscience, Houston, TX, USA, Human TNF-α ELISA kit; Elabscience, Houston, TX, USA and Human oxLDL ELISA kit; Elabscience, Houston, TX, USA, respectively).

### Test breads

The bread developed for the study was a wheat bread, produced with wholemeal soft flour, enriched with HT encapsulated into α-CD (HTB). HT and α-CD were mixed in an aqueous solution in 1:1 molar ratio. The solution was frozen at − 40 °C for 24 h and then lyophilized for approximately 48 h until all moisture had been sublimated. The resulting powder was collected as HT/α-CD inclusion complex. Breads were prepared by adding HT/α-CD complex in the dough mixture. The WWB was a conventional whole wheat bread and was used as a control.

The breads were isocaloric and contained similar macronutrient profile (protein, carbohydrates, fat). The only difference between them was the existence of HT in HTB compared to WWB in which no HT was added.

Table 1 details the nutritional composition of the two tested breads. Nitrogen (protein: Nx6.25) was measured by Kjeldahl (ISO 1871), fat by Soxhlet procedures and total dietary fibers were determined by the AOAC method 991.43. Total HT content was measured by UV–Visible spectroscopy (absorbance at 282 nm).

**Table 1:** Nutritional composition of the two types of bread, each providing 50 g of available carbohydrates

Bread	Energy content (Kcal)	Carbohydrates (g)	Total dietary fiber (g)	Fat (g)	Protein (g)	a-CD (g)	HT (mg)
WB	246	35.2	6.8	4.6	12.5	–	–
HTB	246	35.2	7.3	4.6	12.5	0.5	54

Data are presented per 100 g

WWB whole wheat bread, HTB whole wheat bread enriched with encapsulated hydroxytyrosol (HT)

Total dietary fibers include the amounts of α-cyclodextrin (α-CD) in each bread

### Statistical analysis

The primary endpoint was the comparison of the decrease of HbA_1_c levels between the subjects of the two groups. It was estimated via power calculations that a sample of a total of 60 subjects would allow the detection of a 0.5% greater reduction in HbA_1_c in the intervention group compared to the control group, with a power of 80%. By assuming a 15–20% drop-out rate, 75 volunteers were enrolled and 60 completed the 12-week intervention.

The Kolmogorov–Smirnov test was used to check for normal distribution of the data. Descriptive statistics are presented as mean ± SD and the results as mean ± SEM. A paired sample t-test was used to compare anthropometric and biochemical parameters at the beginning and the end of the intervention in each group. Regarding differences between the two groups, an independent samples t-test was performed. p < 0.05 was considered statistically significant. The SPSS 21.0 statistical software package (IBM, New York, USA) was used for analyses.

## Results

The baseline anthropometric, clinical and biochemical characteristics of the participants are reported in Table 2. The volunteers of the two groups were similar with respect to age (59.2 ± 8.8 vs. 56.6 ± 10.4 years) (mean ± SD), body weight (93.2 ± 18.2 vs. 94.9 ± 16.5 kg), BMI (33.3 ± 5.3 vs. 33.0 ± 4.3 kg/m^2^), waist circumference (111.7 ± 13.3 vs. 112.8 ± 11.1 cm) andHbA_1_c levels (6.9 ± 1.2 vs. 6.8 ± 1.0%). No significant differences in the baseline characteristics between the two groups were observed.

**Table 2:** Anthropometric, clinical and biochemical characteristics of the subjects at baseline and after 12 weeks

Characteristic	Control bread (*n* = 30)			*P* value	Enriched bread (*n* = 30)			*P* value	*P* value (endpoint between groups)	*P* value (change from baseline between groups)
	Baseline	Endpoint	Change from baseline		Baseline	Endpoint	Change from baseline			
Sex (male/female)	13/17	13/17	–	–	16/14	16/14	–	–	–	–
Age (years)	59.2 ± 8.8	59.2 ± 8.8	–	–	56.6 ± 10.4	56.6 ± 10.4	–	–	–	–
Weight (kg)	93.2 ± 18.2	89.4 ± 17.5	− 3.8 ± 2.2	**< 0.001**	94.9 ± 16.5	89.7 ± 15.6	− 5.2 ± 3.0	**< 0.001**	0.927	0.058
BMI (kg/m^2^)	33.3 ± 5.3	31.9 ± 5.0	− 1.3 ± 0.1	**< 0.001**	33.0 ± 4.3	31.2 ± 4.0	− 1.8 ± 0.2	**< 0.001**	0.587	0.090
WC (cm)	111.7 ± 13.3	106.9 ± 12.2	− 4.7 ± 0.4	**< 0.001**	112.8 ± 11.1	107.3 ± 10.0	− 5.5 ± 0.5	**< 0.001**	0.908	0.205
Body fat (%)	38.0 ± 9.0	35.8 ± 9.1	− 2.3 ± 0.3	**< 0.001**	37.1 ± 8.1	33.8 ± 8.8	− 3.3 ± 0.4	**< 0.001**	0.419	**0.038**
Body fat mass (kg)	35.4 ± 12.7	32.0 ± 11.9	–	**< 0.001**	35.5 ± 10.2	30.6 ± 9.8	–	**< 0.001**	0.636	–
Lean mass (kg)	56.2 ± 10.9	55.8 ± 10.7	− 0.4 ± 0.3	0.240	59.6 ± 12.6	59.4 ± 12.4	− 0.2 ± 0.3	0.528	0.255	0.784
SBP (mmHg)	133.5 ± 19.2	126.0 ± 14.5	− 7.4 ± 2.7	**0.011**	130.8 ± 17.9	119.4 ± 13.8	− 11.4 ± 2.6	**< 0.001**	0.074	0.292
DBP (mmHg)	85.2 ± 10.7	82.2 ± 11.3	− 4.5 ± 4.0	**0.048**	82.9 ± 9.7	78.2 ± 7.4	− 11.2 ± 2.6	**0.002**	0.106	0.163
Glucose (mg/dL)	142.3 ± 45.5	123.2 ± 43.4	− 19.1 ± 7.8	**0.021**	132.8 ± 50.2	101.4 ± 19.9	− 31.4 ± 9.9	**0.004**	**0.015**	0.335
HbA_1_c (%)	6.9 ± 1.2	6.4 ± 0.9	− 0.5 ± 0.2	**0.001**	6.8 ± 1.0	6.0 ± 0.6	− 0.8 ± 0.2	**< 0.001**	0.093	0.333
Cholesterol (mg/dL)	172.6 ± 44.3	165.0 ± 34.6	− 7.5 ± 5.5	0.185	167.0 ± 49.3	158.2 ± 44.7	− 8.8 ± 5.6	**0.018**	0.444	0.687
HDL-C (mg/dL)	46.6 ± 12.4	45.7 ± 10.7	− 0.2 ± 1.5	0.399	46.6 ± 13.1	46.7 ± 13.2	+ 0.1 ± 0.8	0.894	0.648	0.930
LDL-C (mg/dL)	93.0 ± 33.1	92.5 ± 30.1	− 0.5 ± 4.7	0.916	95.2 ± 44.5	88.5 ± 40.4	− 6.7 ± 3.8	**0.043**	0.797	0.216
Triacylglycerols (mg/dL)	151.6 ± 61.4	140.9 ± 70.1	− 10.3 ± 7.8	0.201	129.4 ± 52.8	121.0 ± 55.8	− 8.4 ± 5.8	0.158	0.231	0.191
AST (U/L)	19.3 ± 7.3	18.1 ± 7.6	− 1.2 ± 0.9	0.178	18.3 ± 6.3	17.7 ± 5.5	− 0.6 ± 0.7	0.447	0.816	0.580
ALT (U/L)	23.4 ± 12.2	20.5 ± 11.9	− 2.9 ± 1.3	**0.031**	23.1 ± 11.2	19.4 ± 9.1	− 3.7 ± 1.5	**0.017**	0.680	0.657
γ-GT (U/L)	23.7 ± 12.1	21.3 ± 9.7	− 2.4 ± 1.0	**0.027**	24.0 ± 10.0	21.6 ± 8.3	− 2.6 ± 1.3	0.066	0.671	0.991
Urea (mg/dL)	34.7 ± 9.3	36.7 ± 10.8	+ 2.0 ± 1.2	0.120	34.4 ± 9.9	34.4 ± 9.5	+ 0.0 ± 1.5	1.000	0.384	0.323
Creatinine (mg/dL)	0.8 ± 0.2	0.8 ± 0.2	+ 0.0 ± 0.0	0.111	0.8 ± 0.2	0.8 ± 0.2	+ 0.0 ± 0.0	0.126	0.829	0.455
Uric acid (mg/dL)	5.6 ± 1.5	5.8 ± 1.3	+ 0.4 ± 0.2	0.124	5.4 ± 1.6	5.5 ± 1.3	+ 0.1 ± 0.2	0.612	0.376	0.298

Values are expressed as mean ± SD

Paired samples t-test has been performed regarding intra-group changes, while independent samples t-test has been performed regarding inter-group changes

*BMI* body mass index, *WC* waist circumference, *SBP* systolic blood pressure, *DBP* diastolic blood pressure, *HDL*-*C* high-density lipoprotein cholesterol, *LDL*-*C* low-density lipoprotein cholesterol, *AST* aspartate aminotransferase, *ALT* alanine aminotransferase, *γ-GT* γ-glutamyl transferase

Values in bold denote statistical significance at *P*<0.05 level

In Table 2 anthropometric and biochemical measures of the two groups at the end of the 12-week dietary intervention are also presented. Significant differences in body weight, body fat and waist circumference were noted in both control and intervention groups (body weight: 89.4 ± 17.5 vs. 89.7 ± 15.6 kg, body fat: 35.8 ± 9.1 vs. 33.8 ± 8.8%, waist circumference: 106.9 ± 12.2 vs. 107.3 ± 10.0 cm, respectively, *p* < 0.001). There was a greater reduction regarding these parameters in the intervention group between which body fat mass reached statistical significance and weight loss presented marginal significance (*p* = 0.038 and *p* = 0.058, respectively). No changes in lean body mass were observed. Significant decreases were also reported in systolic and diastolic blood pressure, glucose and HbA_1_c levels and concentrations of the AST and ALT enzymes in both groups (*p* < 0.05). With respect to fasting glucose levels there was a significantly greater decrease in the intervention group compared to the control group (101.4 ± 19.9 vs. 123.2 ± 43.4 mg/dL, respectively, *p* = 0.015). A marginally significant difference was also noted in the HbA_1_c levels between the intervention and the control group at the end of the intervention (6.0 ± 0.6 vs. 6.4 ± 0.9%, respectively, *p* = 0.093). Significant reductions of about 9 mg/dL and 7 mg/dL in the intervention group were reported, in total cholesterol and LDL cholesterol levels, respectively. No further significant changes or between-group differences were identified for the biochemical indices.

In Table 3 comparisons in the total energy and macronutrient consumption between the subjects of the two groups at the beginning and the end of the 12-week intervention are depicted. Decreases were observed in both HTB and WWB groups; yet there were no significant differences between them.

**Table 3:** Energy and nutrient consumption of the subjects at baseline and at the end of the intervention

Characteristic	Baseline (*n* = 30)		*P* value	Endpoint (*n* = 30)		*P* value
	Control bread	Enriched bread		Control bread	Enriched bread	
Calorie intake (kcal)	2402.1 ± 532.4	2498.3 ± 450.9	0.453	1933.9 ± 325.4	1971.9 ± 359.0	0.669
Protein intake (g)	89.8 ± 18.8	97.5 ± 22.8	0.161	81.4 ± 15.1	80.6 ± 16.6	0.850
Carbohydrate intake (g)	235.4 ± 71.6	237.8 ± 62.5	0.890	174.4 ± 40.6	185.9 ± 40.2	0.275
Fat intake (g)	123.0 ± 33.3	135.2 ± 39.5	0.199	103.7 ± 22.9	107.4 ± 29.9	0.591

Values are expressed as mean ± SD

Independent samples t-test has been performed regarding inter-group changes

Changes in hormone levels and inflammatory markers are presented in Table 4. No significant differences were there at the beginning of the intervention between the two groups. No significant differences in the levels of insulin, leptin, adiponectin, CRP, oxidized LDL and TNF-α were observed at the control group. At the intervention group, significant reductions in insulin, and TNF-α levels and increase in adiponectin levels (*p* = 0.032, *p* = 0.019 and *p* = 0.002, respectively) and a marginally significant reduction in leptin levels (*p* = 0.081) were observed. A trend towards a greater insulin, TNF-α and adiponectin change (*p* = 0.055, *p* = 0.077 and *p* = 0.096, respectively) in subjects of the HT group was also reported.

**Table 4:** Changes in levels of hormones and inflammatory markers for the two groups

Characteristic	Control bread (*n* = 30)			*P* value	Enriched bread (*n* = 30)			*P * value	*P *value (endpoint between groups)	*P *value (change from baseline between groups)
	Baseline	Endpoint	Change from baseline		Baseline	Endpoint	Change from baseline			
Insulin (μIU/mL)	16.39 ± 1.88	18.11 ± 2.71	+ 1.66 ± 0.39	0.241	15.78 ± 1.56	12.09 ± 1.45	− 4.36 ± 0.72	**0.032**	0.055	**0.008**
Leptin (ng/mL)	26.25 ± 3.52	25.35 ± 2.26	− 0.90 ± 0.53	0.560	27.00 ± 2.26	23.38 ± 1.83	− 3.62 ± 1.01	0.081	0.603	0.286
hs-CRP (mg/L)	5.09 ± 1.13	4.21 ± 0.66	− 1.83 ± 0.78	0.144	3.92 ± 1.13	3.36 ± 0.88	− 0.30 ± 0.41	0.169	0.178	0.090
oxLDL (mU/mL)	95.78 ± 4.12	92.21 ± 3.59	− 3.57 ± 2.64	0.187	103.86 ± 3.83	97.88 ± 3.26	− 5.98 ± 2.09	0.128	0.247	0.606
TNF-α (pg/mL)	0.38 ± 0.07	0.45 ± 0.08	+ 0.07 ± 0.05	0.278	0.47 ± 0.12	0.25 ± 0.07	− 0.22 ± 0.07	**0.019**	0.077	**0.010**
Adiponectin (ng/mL)	8.54 ± 0.39	8.60 ± 0.35	+ 0.06 ± 0.08	0.713	8.47 ± 0.29	9.45 ± 0.35	+ 3.91 ± 0.29	**0.002**	0.096	**0.010**

Values are expressed as mean ± SEM

Paired samples t-test has been performed regarding intra-group changes, while independent samples t-test has been performed regarding inter-group changes

Values in bold denote statistical significance at the *P*<0.05 level

## Discussion

In the present study the effects of a 12-week dietary intervention with whole wheat bread enriched with HT on weight loss, biochemical parameters and markers of inflammation were examined.

Primary outcome of this study was the decrease of HbA_1_c in patients with T2DM. Except for HbA_1_c, diabetes biomarkers such as fasting glucose and insulin were evaluated. Both enriched and conventional whole wheat breads were associated with statistically significant reductions of HbA_1_c, probably due to the adherence to a hypocaloric Mediterranean-based diet. Regarding the comparison between the two groups, a marginal significance in favor of the intervention group was revealed. This finding is in accordance with previous meta-analyses of human interventions with pure polyphenols or polyphenol-rich extracts. Palma-Duran et al. showed that the polyphenol supplementation in T2DM patients was related with a significant reduction of 0.21% in HbA_1_c levels [21]. A similar alteration (0.24% reduction) was also observed in a recent meta-analysis of Raimundo et al. [22]. The revealed trend regarding HT may be due to this polyphenol action at the intestinal level to delay carbohydrate breakdown and glucose absorption. HT is a strong inhibitor of α-glycosidase and presents a mild inhibition against α-amylase [23]. Wheat bread, a staple food daily consumed by various populations across the earth [24], is a main contributor to the postprandial glycaemia as a carbohydrate rich food; therefore, its enrichment with HT could have positive glucose postprandial effects.

Apart from postprandial glycaemia, fasting glycaemia is also strongly correlated with HbA_1_c. In the present study, HTB consumption showed a statistically significant reduction of fasting glucose in comparison with WB reduction. In previous studies, several polyphenols like cocoa, tea or cinnamon polyphenols have been correlated with amelioration of fasting glucose in T2DM or obese subjects [25–27]. Both in vitro and in vivo studies indicate that HT may have insulin like effects on target cells such as adipocytes, hepatocytes or muscle cells improving glucose homeostasis [10]. Regarding insulin levels in this study, HTB contributed to a significant reduction, while WB did not lead to any change. This difference was marginally significant in between-group comparison. Our finding is in accordance with previous in vivo studies in mice [28, 29]. Regarding human interventions, only one study by de Bock et al. has examined the effects of olive leaf extract polyphenols on overweight male subjects for 12 weeks. Except for the improvement in insulin sensitivity, the study revealed a possible increase in pancreatic b-cell secretory capacity after olive polyphenols’ supplementation [30].

All subjects, regardless group, experienced significant weight loss and beneficial changes in waist circumference and blood pressure; however, there was no statistical significance in those parameters between the two groups. This could be explained by the well-established effect of the MD on weight loss and blood pressure [31, 32]. Moreover, a similar decrease in caloric and macronutrient intake, during the 12-week intervention, in both groups was observed. Our finding is in line with previous literature, as no association between HT and better appetite regulation, therefore greater results in weight loss, has been observed in both humans and animal models [30, 33, 34].

Regarding the body fat parameter, both groups experienced significant body fat loss. However, the HTB group achieved greater decrease (14.4%) compared to the WWB group (10.2%). Only few studies have examined the effects of HT supplementation on body composition [33, 35–37]. In accordance with these studies, we observed significant differences in the reduction of fat mass levels between the two groups. Studies in rat models have revealed either prevention of obesity (and increase of fat mass) or decrease in visceral fat levels in obese mice after HT supplementation [35–37]. In a recent study in humans, daily ingestion of gastroresistant capsules containing HT (15 mg of HT/day) for a 3-week period resulted to a decrease in body fat mass percentage [33]. As shown by in vitro models, HT contributes to downregulation of adipogenesis-related genes and therefore protects adipocytes from hyperplasia and hypertrophy. Its consumption improves oxidative adipocyte status and increases its metabolism, by favoring the mitochondria biogenesis [35].

The excessive deposition of body fat is correlated with dysregulation of cytokines produced by adipose tissue such as adiponectin, leptin and TNF-α [38]. Among them, adiponectin seems to play a crucial role as regulator of insulin sensitivity, glucose and lipid metabolism along with its anti-atherogenic, and anti-inflammatory properties [39]. In a previous in vivo study, a correlation between a high quality virgin olive oil, rich in phenolic compounds and an increase in adiponectin levels of overweight/obese subjects was observed [40]. However, this beneficial effect maybe attributed to the synergistic action of the olive oil compounds rather than the one of the individual compounds. Regarding the role of HT, Scoditti et al. showed a beneficial effect on adiponectin production by decreasing TNF-α-induced JNK activation and improving PPARγ expression [41]. In the present study, the intervention group experienced a statistically significant increase in adiponectin levels probably as a result of the marginally greater weight loss.

Moreover, only in HTB group a marginally significant reduction in leptin levels at the end of the intervention was reported. Leptin is an adipocyte-derived hormone with pleiotropic effects on metabolism. Obesity and T2DM are characterized by leptin resistance, which may lead to atherogenic, thrombotic and angiogenic consequences [42]. Data are limited regarding decrease in leptin levels after HT supplementation and are restricted in cellular or animal model studies, showing that HT has a positive impact on the reduction of leptin levels [35, 41]. With respect to the fact that leptin is a pro-inflammatory cytokine similar to IL-6, IL-12 and IL-15 and its production can be induced by other inflammatory mediators such as TNF-α, HT may have led to a decrease in leptin levels directly by exerting an anti-inflammatory action or/and indirectly by decreasing TNF-α levels [43].

As far as blood lipid levels are concerned, significant differences occurred only in the intervention group. Specifically, significant reductions in total cholesterol and LDL cholesterol levels were observed. It is well established that polyphenols have cardioprotective effects and among them, HT is evidenced to be beneficial for preventing CVDs [44]. Studies in rat models have shown that administration of HT (2.5–3 mg/kg of body weight) leads to significant decreases in the serum levels of total cholesterol, triglycerides and LDL cholesterol and increase in HDL cholesterol [45, 46]. Results in clinical trials addressed to human volunteers are controversial. Covaset al. in a crossover study with healthy men found no changes in total or LDL cholesterol 3 weeks post intervention with olive oil enriched in HT [47]. In accordance with our findings, a study in patients with metabolic syndrome, who consumed daily for 8 weeks either a capsule containing 10.82 mg of monacolins from red yeast rice and 9.32 mg of HT from olive oil or a placebo capsule, has shown significant decreases of total and LDL cholesterol in the intervention group. Significant reduction in oxidized LDL has also been reported. The decreases were greater in comparison with our study; however, the observed effects may be attributed to an additional or synergistic effect of the combination of the two components as red yeast rice is well known for its lipid lowering effect [48].

Regarding oxidized LDL, our study has shown a slight decrease (5.8%), which did not reach statistical significance, a finding that agrees with Colica et al. [33]. A previous study, in which 40 mL of olive oils with different phenolic contents; low (2.7 mg/kg), moderate (164 mg/kg) or high (366 mg/kg) were consumed by healthy male volunteers, has shown a reduction in the degree of LDL oxidation proportionally to the phenolic content of the olive oil administered [49]. In a few other studies in human volunteers reductions in oxidized LDL levels after HT supplementation have also been reported [50–52]. However, it is worth noting that three of them examined the postprandial levels of this parameter, which may indicate an acute impact that needs to be further investigated in the long-term [49–51]. Moreover, in the majority of these studies, olive oil and not isolated HT was provided; thus, the positive impact on oxidized LDL levels may be attributed to the synergistic effects of olive oil bioactive compounds.

The long-term consumption of HTB led to attenuation of TNF-α levels while no effect regarding CRP was reported. The positive olive oil effect on inflammation is well recognized. Carmago et al. proposed an interaction of olive polyphenols when consumed as olive oil with NF-κB/MAPK/AP-1 signaling pathways, which results to downregulation of the inflammatory response of the peripheral blood mononuclear cells (PBMCs) [53]. Although these results provide evidence towards the beneficial effect of a polyphenol rich olive oil on transcription level in humans, they are not revealing whether this is attributed to the action of one compound or a synergistic effect [53]. Particularly for HT, it has been associated with anti-inflammatory effects [38]. Evidence derived from an intervention study in rats is in line with the decrease of plasma TNF-α and IL-1β levels induced by HT supplementation [54]. With respect to human studies, even though HT leads to positive effects on plasma cytokine levels, the results are not constant. Lockyer et al. reported that long term administration of olive leaf extract on pre-hypertensive males decreased IL-8, yet it had no effect on other inflammation markers [55]. Another intervention by Mosca et al. in children with non-alcoholic fatty liver disease referred that the treatment with HT and vitamin E led to amelioration in inflammation mainly due to alteration in IL-10 levels, while TNF-αdecreased in both intervention and placebo group [56]. Conclusively, since the circulating cytokine levels highly vary among individuals; more studies with greater sample size are needed in order reliable conclusions to be drawn [57].

Summing up, the enrichment of bread with HT in terms of a balanced energy-restricted diet contributed to great results regarding fasting glucose, insulin and HbA_1_c levels in patients with overweight/ obesity and T2DM. It also led to significant reductions in blood lipid levels and markers of inflammation. However, the findings of this study have to be seen in light of its limitations. The sample size and the duration of this study may not be sufficient for the detection of alterations in some hormonal and oxidative stress markers. Moreover, this is the first study to examine the metabolic effects of a food containing encapsulated HT rather than a HT supplement. Factors such as the food matrix or the consumption in combination with other foods may also play a role in the bioavailability of HT. The incorporation of functional ingredients in foods like bread, which are highly consumed on a daily basis all over the world, is a strategy of great importance, since it could ameliorate the nutritional quality of foods and make them a great option especially for subjects who do not follow the traditional MD and do not consume olive oil on a daily basis. It could also benefit subjects with T2DM or/and overweight/obesity for whom rigorous control of body weight is crucial.

## Data Availability

Raw data that support the findings of this study can be provided by the corresponding author upon reasonable request.
